# Treatment of a Cold

**Published:** 1890-02

**Authors:** 


### Association News—Preparations for the Forty-first Annual Meeting.

*To the Editor* :—I write to state that arrangements for the next meeting of the American Medical Association are being made rapidly and satisfactorily by our several committees. The Committee on Halls and Hotels have completed their work. The general meetings will be held in our largest and finest theatre, the Vendome. A splendid hall has been procured for the meeting of every section, and all of these are within two minutes' walk of the Vendome.

The Committee on Entertainments will do their duty, and will have in store much pleasure for all who may attend; but the entertainments will be so arranged as not to conflict with either general or sectional meetings.

Our hotels and boarding houses can accommodate as many as will come. May we not hope for two thousand at least.

The Committee on Reception will meet all incoming trains, and will welcome all who come, and aid them in securing places for rest and refreshment.

The charges at the hotels will be from $1.50 to $5 per day. There are only a few rooms, and in only one hotel, the Duncan, at $5. Rooms can be engaged beforehand by communicating

